# Half-thickness discretized format for simulating compressible delay interbed

**DOI:** 10.1038/s41598-025-14712-7

**Published:** 2025-08-11

**Authors:** Shangqi Han, Chuiyu Lu, Wen Lu, Qingyan Sun, Chu Wu

**Affiliations:** 1 State Key Laboratory of Simulation and Regulation of Water Cycle in River Basin, China Institute of Water Resources and Hydropower Research, Beijing, 100038 China; 2 Department of Water Resources, China Institute of Water Resources and Hydropower Research (IWHR), Beijing, 100038 China

**Keywords:** Subsidence simulation, Compressible delay interbed, CSUB package, Principle of geostress variation, Half-thickness discretization format, Engineering, Mathematics and computing, Solid Earth sciences

## Abstract

The simulation of compressible delay interbed is an important component of ground subsidence modeling. Currently, the most widely used groundwater simulation software, MODFLOW, has two modules: SUB and CSUB. While both can simulate compressible delay interbed using the one-dimensional head diffusion equation, they differ in approach. The SUB module relies on the principle of head change, while the CSUB module is based on the principle of geostress variation, addressing the shortcomings of the SUB module when simulating ground subsidence in unconfined aquifers. When based on the principle of head change, the effective stress acting on the top and bottom of the interbed is the same, leading to symmetric vertical consolidation and using half-thickness discretization. In contrast, the CSUB module is based on geostress variation, the effective stress acting on the top and bottom of the interbed is not the same, which results in asymmetric consolidation and requires full-thickness discretization. With the same vertical discretization interval for the compressible delay interbed, the computational workload and memory requirements are approximately twice that of the SUB module. Based on the characteristic of linear distribution of geostress in the vertical direction of the compressible delay interbed, this paper proposes a half-thickness discretization format under the principle of geostress variation, which can significantly improve simulation efficiency and reduce memory requirements. To validate this approach, three cases were tested with different numbers of discretization units, different interbed thicknesses, different heads, and different vertical hydraulic conductivities. A comprehensive comparison was made between the half-thickness discretization format and the full-thickness discretization format of the CSUB module. The differences in computation time and memory usage between the two methods were then analyzed. The results show that the maximum difference in the interbed water release between the half-thickness method and the CSUB module is less than 0.4%, indicating good accuracy. Additionally, the half-thickness method reduced computation time by 46.2303% and memory usage by 13.6364% in the tested cases, demonstrating significant computational advantages. This study provides an efficient and feasible technical approach for large-scale, high-precision land subsidence modeling.

## Introduction

Ground subsidence is a downward movement caused by the consolidation and compression of underground loose layers, threatening the sustainable development of the natural environment and the economy^1,2^. Quantitative research on ground subsidence is a key issue in formulating protection policies and restoring the environment^3,4^. The continuous decline of groundwater levels is often a major cause of ground subsidence^5–7^. To address this issue, mitigation measures such as water source substitution and artificial groundwater recharge have been widely implemented to alleviate the risk of land subsidence caused by excessive groundwater extraction^8–10^. Numerical simulation methods based on physical mechanisms can take into account the interaction between groundwater levels and ground subsidence, making them an important tool for studying and predicting the evolution of ground subsidence patterns^11–14^.

**Fig. 1:**
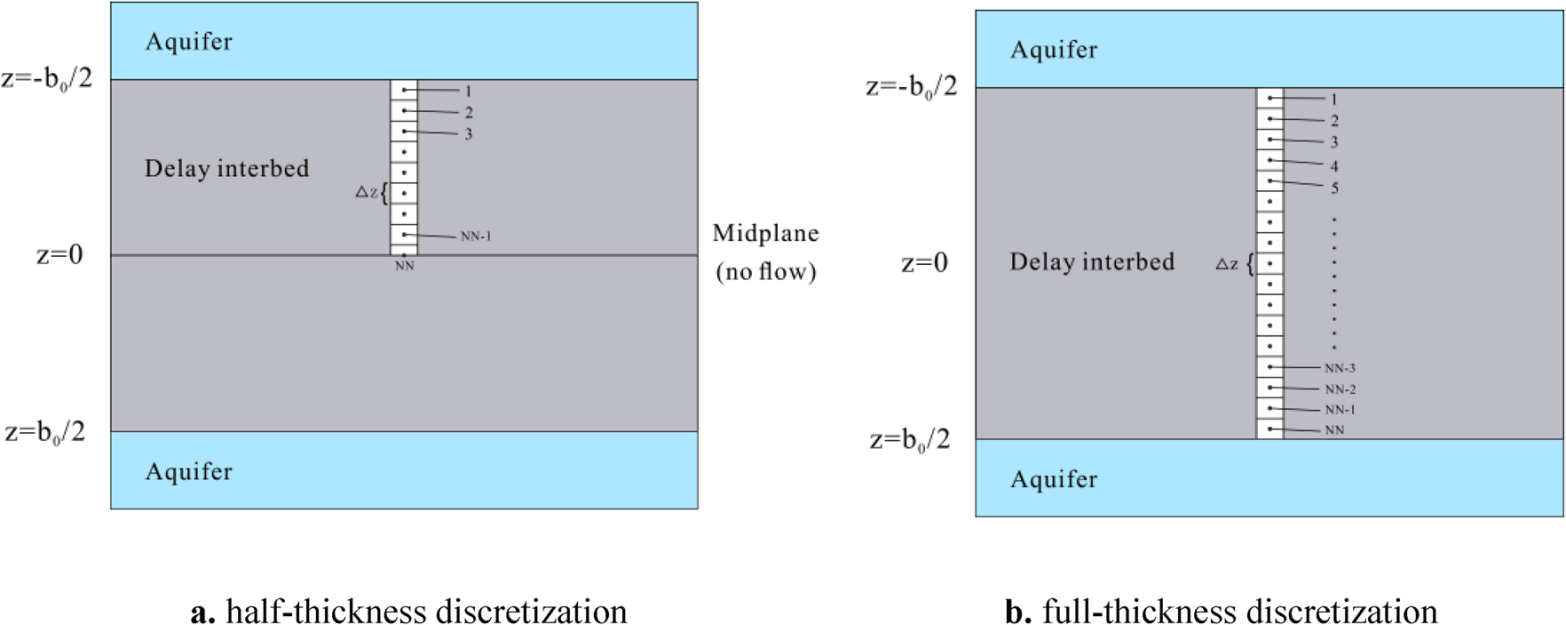
One-dimensional vertical discretization of a compressible delay interbed (**a** half-thickness discretization;** b** full-thickness discretization).

Since 1991, groundwater models represented by MODFLOW have developed the Interbed Storage Package 1 (IBS1)^15^. Subsequently, simulation software packages for ground subsidence, such as SUB, SUB-WT, and CSUB, were developed. Among them, the SUB package was the first to introduce a one-dimensional vertical head diffusion equation to address the drainage time lag issue in interbeds^16^, thereby enabling the simulation of compressible delay interbed. In such interbeds, the hydraulic equilibrium time between the internal head and the surrounding aquifer is significantly longer than the time step used in the model. As a result, the head changes within the interbed lag behind those in the aquifer, and these interbeds are therefore referred to as compressible delay interbeds. The SUB package is based on the principle of head change, which assumes that the change in effective stress at any position within the aquifer is equal to the negative value of the change in head^17^. Therefore, when simulating compressible delay interbed, the effective stress acting on the top and bottom of the interbed is the same. By utilizing the principle of symmetry, the diffusion equation can be solved by discretizing half of the thickness of the compressible delay interbed (as shown in Fig. 1), which improves simulation efficiency and reduces memory requirements. This method is referred to as the half-thickness discretization format. The subsequent ground subsidence simulation software packages, such as SUB-WT and CSUB, are all based on the principle of changes in geostress^18,19^. However, SUB-WT can only simulate no-delay interbed. In contrast, the CSUB package integrated into the latest version of MODFLOW 6 incorporates the complete functionality for simulating coarse-grained media within aquifers, no-delay interbeds, compressible delay interbeds, and the compaction and release of pore water in aquifers. Compared to the SUB module, the subsidence simulation in CSUB is more realistic when dealing with unconfined aquifers, as the impact of fluctuations in the groundwater level on geostress can be reflected in CSUB. In contrast, the SUB module, which is based on the principle of head change, cannot account for this effect, often resulting in significantly overestimated subsidence values^17,19^. However, when based on the principle of changes in geostress, the effective stress at any position within the aquifer is equal to the total geostress at that location minus the pore water pressure. Since total geostress increases with depth, the effective stress acting on the top and bottom of a compressible delayed interbed differs. Generally, a full-thickness discretization format is required (as shown in Fig. 1). As a result, under the same vertical discretization spacing for the compressible delay interbed, the computational workload and memory requirements for CSUB when simulating compressible delay interbed are approximately twice that of SUB. This significantly impacts model run time and, when the simulation scale is large, places higher demands on the memory capacity of the computing device.

To address the above issues, this paper proposes a half-thickness discretization format for compressible delay interbeds under the principle of changes in geostress. This method can significantly reduce the computational workload and memory requirements, similar to the SUB module, while also ensuring a comparable level of simulation accuracy. Currently, this method is integrated into the COMUS model (C++ Object-Oriented Model for Underground Water Simulation) developed by the China Institute of Water Resources and Hydropower Research^20,21^. The subsequent sections of the paper provide a detailed introduction to the computational principles of this method and establish three representative examples that consider the effects of different numbers of discretization units, different interbed thicknesses, different heads, and different vertical hydraulic conductivities. These examples are compared with the full-thickness discretization method of the CSUB package to validate the applicability of the newly derived method. Finally, the improvements in computational efficiency were quantitatively analyzed.

## Methods

In ground subsidence simulations, the interbed is considered to be delay interbed if the equilibrium time of the head within interbed is significantly longer than the time step used in the simulation. When the horizontal extent of the interbed is significantly greater than its thickness, and the horizontal hydraulic conductivity of the aquifer is much greater than the vertical hydraulic conductivity of the interbed, the flow of water within the interbed can be considered vertical. Based on the principle of changes in geostress, the compaction and release of pore water in the interbed can be expressed using the following one-dimensional head diffusion equation:

1 $$K_{v}{^\prime} \frac{{\partial^{2} h}}{{\partial z^{2} }} = - \frac{{S_{s} }}{{\gamma_{w} }}\frac{{\partial \sigma{^\prime} }}{\partial t}$$

where: $K_{v}{^\prime}$ is the vertical hydraulic conductivity of the compressible delay interbed (*L/T*); $h$ is the head within the compressible delay interbed (*L*); $z$ is the vertical spatial coordinate (*L*); $S_{s}$ is the specific storage(-);$t$ is time (*T*);$\gamma_{w}$ is the unit weight of water (*M/L2/T2*); $\sigma{^\prime}$ is the effective stress (*M/L/T2*).

In the half-thickness discretization format (Fig. 1a), considering water entering the unit as positive, the numerical discretization of the partial differential equation is performed according to Eq. (1). For any arbitrary *m*-th calculation period, the water balance equation at node unit *i*=1 is given by:

2 $$\begin{gathered} K_{v}{^\prime} \frac{{h_{j}^{m} - h_{1}^{m} }}{0.5\Delta z} + K_{v}{^\prime} \frac{{h_{2}^{m} - h_{1}^{m} }}{\Delta z} = - \frac{{S_{sk,1}^{m} }}{\Delta t}\left( {\frac{{\sigma{^\prime}_{1}^{m} }}{{\gamma_{w} }} - \frac{{\sigma{^\prime}_{c,1}^{m - 1} }}{{\gamma_{w} }}} \right)\Delta z \hfill \\ \qquad - \frac{{S_{ske,1}^{m} }}{\Delta t}\left( {\frac{{\sigma{^\prime}_{c,1}^{m - 1} }}{{\gamma_{w} }} - \frac{{\sigma{^\prime}_{1}^{m - 1} }}{{\gamma_{w} }}} \right)\Delta z \hfill \\ \end{gathered}$$

where: *∆z* is the vertical discretization distance between the centers of two node units in the equivalent interbed (*L*); $\Delta t$ is the time step (*T*); $S_{sk,1}^{m}$ is the specific storage to be selected at node unit 1 for the *m*-th calculation period (1*/L*); $h_{j}^{m}$ is the head of the groundwater cell *j* at the end of the *m*-th calculation period (assuming the equivalent interbed is located above this cell) (*L*); $h_{1}^{m}$ is the head at node unit 1 at the end of the *m*-th calculation period (*L*); $h_{2}^{m}$ is the head at node unit 2 at the end of the *m*-th calculation period (*L*); $S_{sk,1}^{m}$ is the specific storage to be selected at node unit 1 during the *m*-th calculation period (*1/L*); $\frac{{\sigma{^\prime}_{1}^{m} }}{{\gamma_{w} }}$ is the effective stress acting on node unit 1 at the end of the *m*-th calculation period (measured in terms of water column height) (*L*); $\frac{{\sigma{^\prime}_{c,1}^{m - 1} }}{{\gamma_{w} }}$ is the preconsolidation stress on node unit 1 at the end of the (*m*−1)-th computation period (measured in terms of water column height) (*L*); $S_{ske,1}^{m}$ is the elastic specific storage at node unit 1 during the *m*-th calculation period (*1/L*); $\frac{{\sigma{^\prime}_{1}^{m - 1} }}{{\gamma_{w} }}$ is the effective stress acting on node unit 1 at the end of the (*m*−1)-th calculation period (measured in terms of water column height) (*L*).

The effective stress acting on node unit 1 at the end of the *m*-th calculation period in Eq. (2) is calculated as:

3 $$\frac{{\sigma{^\prime}_{1}^{m} }}{{\gamma_{w} }} = \frac{{\sigma_{1}^{m} }}{{\gamma_{w} }} - (h{^\prime}_{1}^{m} - z_{1} )$$

where: $\frac{{\sigma_{1}^{m} }}{{\gamma_{w} }}$ is the total geostress acting on node unit 1 at the end of the *m*-th calculation period (measured in terms of water column height) (*L*); $z_{1}$ is the elevation of the center of node unit 1 (*L*).

By combining Eqs. (2) and (3), we obtain:

4 $$\begin{gathered} - \left( {\frac{{3K_{v}^{'} }}{{\Delta z}} + \frac{{\Delta z}}{{\Delta t}}S_{{sk,1}}^{m} } \right)h_{1}^{m} + \frac{{K_{v}^{'} }}{{\Delta z}}h_{2}^{m} = - \frac{{\Delta z}}{{\Delta t}}\left[ {S_{{sk,1}}^{m} \left( {\frac{{\sigma _{1}^{m} }}{{\gamma _{w} }} + z_{1} } \right) - } \right. \hfill \\ \left. {\qquad \left(S_{{sk,1}}^{m} - S_{{ske,1}}^{m} \right)\frac{{\sigma _{{c,1}}^{{{^\prime} m - 1}} }}{{\gamma _{w} }} - S_{{ske,1}}^{m} \frac{{\sigma _{1}^{{{^\prime} m - 1}} }}{{\gamma _{w} }}} \right] - \frac{{2K_{v}^{'} }}{{\Delta z}}h_{j}^{m} \hfill \\ \end{gathered}$$

Similarly, for the node units where 1<*i*<*NN*, their difference equations can be established as follows:

5 $$\begin{gathered} K_{v}{^\prime} \frac{{h_{i - 1}^{m} - h_{i}^{m} }}{\Delta z} + K_{v}{^\prime} \frac{{h_{i + 1}^{m} - h_{i}^{m} }}{\Delta z} = - \frac{{S_{sk,i}^{m} }}{\Delta t}\left( {\frac{{\sigma{^\prime}_{i}^{m} }}{{\gamma_{w} }} - \frac{{\sigma{^\prime}_{c,i}^{m - 1} }}{{\gamma_{w} }}} \right)\Delta z \hfill \\ \qquad - \frac{{S_{ske,i}^{m} }}{\Delta t}\left( {\frac{{\sigma{^\prime}_{c,i}^{m - 1} }}{{\gamma_{w} }} - \frac{{\sigma{^\prime}_{i}^{m - 1} }}{{\gamma_{w} }}} \right)\Delta z \hfill \\ \end{gathered}$$

This simplifies to:

6 $$\begin{gathered} \frac{{K_{v}^{'} }}{{\Delta z}}h_{{i - 1}}^{m} - \left( {\frac{{2K_{v}^{'} }}{{\Delta z}} + \frac{{\Delta z}}{{\Delta t}}S_{{sk,i}}^{m} } \right)h_{i}^{m} + \frac{{K_{v}^{'} }}{{\Delta z}}h_{{i + 1}}^{m} = - \frac{{\Delta z}}{{\Delta t}}\left[ {S_{{sk,i}}^{m} \left( {\frac{{\sigma _{i}^{m} }}{{\gamma _{w} }} + z_{i} } \right) - } \right. \hfill \\ \left. {\qquad \left(S_{{sk,i}}^{m} - S_{{ske,i}}^{m} \right)\frac{{\sigma _{{c,i}}^{{{^\prime} m - 1}} }}{{\gamma _{w} }} - S_{{skei}} \frac{{\sigma _{i}^{{{^\prime} m - 1}} }}{{\gamma _{w} }}} \right] \hfill \\ \end{gathered}$$

Finally, for the node unit *NN*, establishing its difference equation, noting that the thickness of node unit *NN* is only *∆z*/2, we have:

7 $$\begin{gathered} K_{v}{^\prime} \frac{{h_{NN - 1}^{m} - h_{NN}^{m} }}{\Delta z} = - \frac{{S_{sk,NN}^{m} }}{\Delta t}\left( {\frac{{\sigma{^\prime}_{NN}^{m} }}{{\gamma_{w} }} - \frac{{\sigma{^\prime}_{c,NN}^{m - 1} }}{{\gamma_{w} }}} \right)\frac{\Delta z}{2} \hfill \\ \qquad - \frac{{S_{ske,NN}^{m} }}{\Delta t}\left( {\frac{{\sigma{^\prime}_{c,NN}^{m - 1} }}{{\gamma_{w} }} - \frac{{\sigma{^\prime}_{NN}^{m - 1} }}{{\gamma_{w} }}} \right)\frac{\Delta z}{2} \hfill \\ \end{gathered}$$

This simplifies to:

8 $$\begin{gathered} \frac{{K_{v}{^\prime} }}{\Delta z}h_{NN - 1}^{m} - \left( {\frac{{K_{v}{^\prime} }}{\Delta z} + \frac{\Delta z}{{2\Delta t}}S_{sk,NN}^{m} } \right)h_{NN}^{^{^\prime}m} = - \frac{\Delta z}{{2\Delta t}}\left[ {S_{sk,NN}^{m} \left( {\frac{{\sigma_{NN}^{m} }}{{\gamma_{w} }} + z_{NN} } \right) } \right. \hfill \\ \qquad \left.- {\left( {S_{sk,NN}^{m} - S_{ske,NN}^{m} } \right)\frac{{\sigma_{c,NN}^{{^\prime} m - 1} }}{{\gamma_{w} }} - S_{skeNN} \frac{{\sigma_{NN}^{{^\prime} m - 1} }}{{\gamma_{w} }}} \right] \hfill \\ \end{gathered}$$

By combining the equations of the various node units, namely (4), (6), and (8), we can obtain the matrix equation:

9 $$[A]^{m} [h]^{m} = [r]^{m}$$

where: [*A*]*m* is a tridiagonal symmetric matrix of size *NN*×*NN*; [h]*m* is the one-dimensional vector of unknown water head values with *NN* elements; [*r*]*m* is the one-dimensional vector of known values with *NN* elements. This matrix equation is a symmetric tridiagonal system and can be solved using the Gaussian elimination method.

The elements of the matrix equation are as follows:

10 $$S_{sk,i}^{m} = \left\{ {\begin{array}{*{20}l} {S_{ske,i}^{m} \quad if:\frac{{\sigma{^\prime}_{i}^{m} }}{{\gamma_{w} }} \le \frac{{\sigma{^\prime}_{c,i}^{m - 1} }}{{\gamma_{w} }}} \hfill \\ {S_{skv,i}^{m} \quad if:\frac{{\sigma{^\prime}_{i}^{m} }}{{\gamma_{w} }} > \frac{{\sigma{^\prime}_{c,i}^{m - 1} }}{{\gamma_{w} }}\quad } \hfill \\ \end{array} for:1 \le i \le NN} \right.$$

11 $$A_{ij}^{m} = \frac{{K_{v}^{\prime } }}{\Delta }\quad for\,:i \ne j$$

12 $$A_{11}^{m} = - \frac{{3K_{v}{^\prime} }}{\Delta z} - \frac{\Delta z}{{\Delta t}}S_{sk,1}^{m}$$

13 $$A_{ii}^{m} = - \frac{{2K_{v}{^\prime} }}{\Delta z} - \frac{\Delta z}{{\Delta t}}S_{sk,i}^{m} \quad for:1 < i < NN$$

14 $$A_{{NN{\kern 1pt} NN}}^{m} = - \frac{{K_{v}{^\prime} }}{\Delta z} - \frac{\Delta z}{{2\Delta t}}S_{sk,NN}^{m}$$

15 $$\begin{aligned} r_{1}^{m} = & - \frac{{\Delta z}}{{\Delta t}}\left[ {S_{{sk,1}}^{m} \left( {\frac{{\sigma _{1}^{m} }}{{\gamma _{w} }} + z_{1} } \right) - \left( {S_{{sk,1}}^{m} - S_{{ske,1}}^{m} } \right)\frac{{\sigma _{{c,1}}^{{{^\prime} m - 1}} }}{{\gamma _{w} }} } \right. \\ & \left. -{S_{{ske,1}}^{m} \frac{{\sigma _{1}^{{{^\prime} m - 1}} }}{{\gamma _{w} }}} \right] - \frac{{2K_{v}^{'} }}{{\Delta z}}h_{j}^{m} \\ \end{aligned}$$

16 $$\begin{aligned} r_{i}^{m} = & - \frac{{\Delta z}}{{\Delta t}}\left[ {S_{{sk,i}}^{m} \left( {\frac{{\sigma _{i}^{m} }}{{\gamma _{w} }} + z_{i} } \right) - (S_{{sk,i}}^{m} - S_{{ske,i}}^{m} )\frac{{\sigma _{{c,i}}^{{{^\prime} m - 1}} }}{{\gamma _{w} }} } \right. \\ & \left. -{S_{{skei}} \frac{{\sigma _{i}^{{{^\prime} m - 1}} }}{{\gamma _{w} }}} \right]\quad for:1 < i < NN \\ \end{aligned}$$

17 $$\begin{aligned} r_{{NN}}^{m} & = - \frac{{\Delta z}}{{2\Delta t}}\left[ {S_{{sk,NN}}^{m} \left( {\frac{{\sigma _{{NN}}^{m} }}{{\gamma _{w} }} + z_{{NN}} } \right) - \left(S_{{sk,NN}}^{m} - S_{{ske,NN}}^{m} \right)\frac{{\sigma _{{c,NN}}^{{{^\prime} m - 1}} }}{{\gamma _{w} }}} \right. \\ & \quad \left. { - S_{{skeNN}} \frac{{\sigma _{{NN}}^{{{^\prime} m - 1}} }}{{\gamma _{w} }}} \right] \\ \end{aligned}$$

The difference between the half-thickness discretization format in this article and the full-thickness discretization format of CSUB is related not only to the spatial discretization of node units but also to the computation of specific storage for interbed.

In CSUB, the specific storage for different compressible delay interbed node units during each period is calculated the following formula:

18 $$\left\{ {\begin{array}{*{20}c} {S_{skv,i}^{m} = \frac{{0.434C_{c} \gamma_{w} }}{{\sigma{^\prime}_{i}^{m} \left( {1 + e_{0} } \right)}}} \\ {S_{ske,i}^{m} = \frac{{0.434C_{r} \gamma_{w} }}{{\sigma{^\prime}_{i}^{m} \left( {1 + e_{0} } \right)}}} \\ \end{array} } \right.$$

where: $C_{c}$ and *C*_*r*_ are the dimensionless compression and recompression indices of the compressible delay interbed (-); *e*_0_ is the initial void ratio of the compressible delay interbed (-).

The half-thickness discretization format in this article employs the following calculation formula:

19 $$\left\{ {\begin{array}{*{20}c} {S_{skv,i}^{m} = \frac{{0.434C_{c} \gamma_{w} }}{{\sigma{^\prime}_{B,i}^{m} \left( {1 + e_{0} } \right)}}} \\ {S_{ske,i}^{m} = \frac{{0.434C_{r} \gamma_{w} }}{{\sigma{^\prime}_{B,i}^{m} \left( {1 + e_{0} } \right)}}} \\ \end{array} } \right.$$

where the corrected effective stress $\sigma{^\prime}_{B,i}^{m}$ at node unit *i* is calculated using the formula:

20 $$\frac{{\sigma{^\prime}_{B,i}^{m} }}{{\gamma_{w} }} = \frac{{\sigma_{B}^{m} }}{{\gamma_{w} }} - (h{^\prime}_{i}^{m} - z_{B} )$$

where: $z_{B}$ is the vertical position at the center of the compressible delay interbed (*L*); $\frac{{\sigma_{B}^{m} }}{{\gamma_{w} }}$ is the total geostress at the center of the compressible delay interbed (measured in water column height) (*M/L2/T2*).

The mathematical principle behind this correction formula can be explained as follows. For the same compressible delay interbed, assuming that the discretization spacing of the node units is equal in both the half-thickness and full-thickness discretization formats, the half-thickness format has *NN* node units, while the full-thickness format has 2*NN* − 1 node units. For the *i*-th node in the half-thickness discretization format, we expect its specific storage to be the average of the specific storage of the *i*-th and (2*NN* − 1 − *i*)-th node units in the full-thickness discretization format. To achieve this, we calculate the average effective stress $\frac{{\overline{\sigma }{^\prime}_{i,2NN - 1 - i} }}{{\gamma_{w} }}$ of the *i*-th and (2*NN* − 1 − *i*)-th node units, which is given by:

21 $$\begin{gathered} \frac{{\bar{\sigma }^{'} _{{i,2NN - 1 - i}} }}{{\gamma _{w} }} = \left[ {\frac{{\sigma _{i}^{m} }}{{\gamma _{w} }} - (h_{i}^{{{^\prime} m}} - z_{i} ) + \frac{{\sigma _{{2NN - 1 - i}}^{m} }}{{\gamma _{w} }} - \left( {h_{{2NN - 1 - i}}^{{{^\prime} m}} - z_{{2NN - 1 - i}} } \right)} \right]/2 \hfill \\ \qquad = \left( {\frac{{\sigma _{i}^{m} }}{{\gamma _{w} }} + \frac{{\sigma _{{2NN - 1 - i}}^{m} }}{{\gamma _{w} }}} \right)/2 - \left[ {(h_{i}^{{{^\prime} m}} + h_{{2NN - 1 - i}}^{{{^\prime} m}} )/2 - (z_{i} + z_{{2NN - 1 - i}} )/2} \right] \hfill \\ \end{gathered}$$

Since the total geostress is linearly distributed vertically within the compressible delay interbed, and the distances from the *i*-th and (2*NN* − 1 − *i*)-th node units to the center of the compressible delay interbed are equal, we refer to the center position of the compressible delay interbed as follows:

22 $$\left\{ {\begin{array}{*{20}l} {\left(\frac{{\sigma_{i}^{m} }}{{\gamma_{w} }} + \frac{{\sigma_{2NN - 1 - i}^{m} }}{{\gamma_{w} }}\right)/2 = \frac{{\sigma_{B}^{m} }}{{\gamma_{w} }}} \hfill \\ {(z_{i} + z_{2NN - 1 - i} )/2 = z_{B} } \hfill \\ \end{array} } \right.$$

By substituting Eq. (22) into Eq. (21), we can see that:

23 $$\frac{{\overline{\sigma }{^\prime} }}{{\gamma_{w} }} = \frac{{\sigma_{B}^{m} }}{{\gamma_{w} }} - \left[(h{^\prime}_{i}^{m} + h{^\prime}_{2NN - 1 - i}^{m} )/2 - z_{B} \right]$$

In the half-thickness discretization format, the hydraulic head at the *i*-th node unit is approximately equal to the average of the hydraulic heads at the *i*-th and (2*NN* − 1 − *i*)-th node units in the full-thickness discretization format. Therefore, comparing Eqs. (21) and (23), $\frac{{\sigma{^\prime}_{B,i}^{m} }}{{\gamma_{w} }} \approx \frac{{\overline{\sigma }{^\prime}_{i,2NN - 1 - i} }}{{\gamma_{w} }}$ , we find that it aligns with our homogenization expectations.

By solving the matrix equation (Eq. 9), we can simulate the compaction/storage process of the node units in the compressible delay interbed. Since the number of variables in the system of equations is only half that of the full-thickness discretization method, the simulation is more efficient and requires less memory. However, based on the principle of geostress variation, there will be a slight shift between the zero-flux surface within the compressible delay interbed and the center position of the compressible delay interbed. Therefore, the half-thickness discretization format presented in this paper is an approximation of the full-thickness discretization format. Subsequent test cases demonstrate that the simulation deviations between the half-thickness and full-thickness discretization formats remain minimal in practical applications, ensuring a comparable level of accuracy.

## Test simulations

**Fig. 2:**
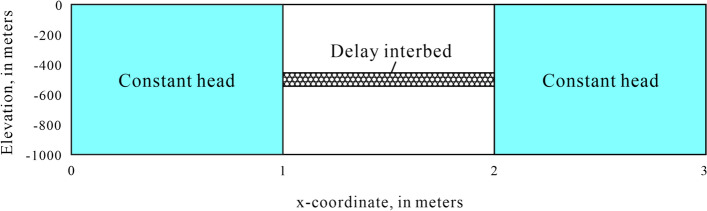
Model setup.

To test the accuracy of the half-thickness discretization calculation method and compare the differences between the two approaches, the tests was conducted based on example 1 from the MODFLOW-SUB documentation^17^ in conjunction with the CSUB example 2 described in the MODFLOW 6 documentation. This example simulates the drainage process of a thick compressible delay interbed caused by a gradual decline of the hydraulic head in the aquifer. The simulation consists of one stress period, with a total duration of 1000 days. It is divided into 100 computation intervals of varying lengths using a time step multiplier of 1.05. The model structure is illustrated in Fig. 2. The model grid consists of 1 layer, 1 row, and 3 columns. The top elevation of the model is 0 m, while the bottom elevation is -1000 m, with both the row and column widths set to 1 m. A compressible delay interbed is situated in the middle cell, where the hydraulic head values of the constant head cell on both sides are set to 0 m. The hydraulic conductivity of the middle cell is set to 1.0E +6 to ensure that the hydraulic head value is also 0 m. Detailed model parameters are provided in Table 1.

**Table 1:** Model parameters.

Parameter	Value
Starting head (m)	0
Specific gravity of moist soils (unitless)	1.7
Specific gravity of saturated soils (unitless)	2
Elastic specific storage of interbed (1/m)	1.0e−5
Inelastic specific storage of interbed (1/m)	1.0e−2
Interbed porosity (unitless)	0.45

To provide a more comprehensive comparison of differences, three cases are defined based on variations in certain parameters of compressible delay interbed. The specific parameters are shown in Table 2. In Case 1, the thickness of the compressible delay interbed is set to three scenarios: 10 m, 50 m, and 100 m, to test the impact of different thicknesses on the calculation results and to explore the applicability of the half-thickness discretization method. In this case, the initial hydraulic head for the compressible delay interbed is set at 10 m, with the initial preconsolidation head equal to the initial hydraulic head. Considering that the number of discretization units and the hydraulic head can also affect the accuracy of the calculation results, Case 2 increases the number of discretization units to 1001, with the initial hydraulic head set at 100 m. Such extreme conditions are generally not encountered in practical scenarios, making this case suitable for validating the reliability of the half-thickness discretization method under extreme conditions. Case 3 sets up three scenarios based on different vertical hydraulic conductivities, which are set to 2.5e−06 m/d, 0.00025 m/d, and 0.025 m/d. All other parameters remain the same as in case 2. This case aims to test the differences between the two methods under varying rates of hydraulic head dissipation within the compressible delay interbed.

**Table 2:** Parameter settings for each test case of the compressible delay interbeds.

Scenarios	Method	Node count	Starting head (m)	Difference between preconsolidation head and initial head(m)	Thickness (m)	K_v_(m/d)
Case 1	Full-thickness discretization method	19	10	0	10,50,100	0.001
	Half-thickness discretization Method	10	10	0	10,50,100	0.001
Case 2	Full-thickness discretization method	1001	100	90	100	0.001
	Half-thickness discretization method	501	100	90	100	0.001
Case 3	Full-thickness discretization method	1001	100	90	100	2.5e−06, 0.00025, 0.025
	Half-thickness discretization method	501	100	90	100	2.5e−06, 0.00025, 0.025

### Case 1

**Fig. 3:**
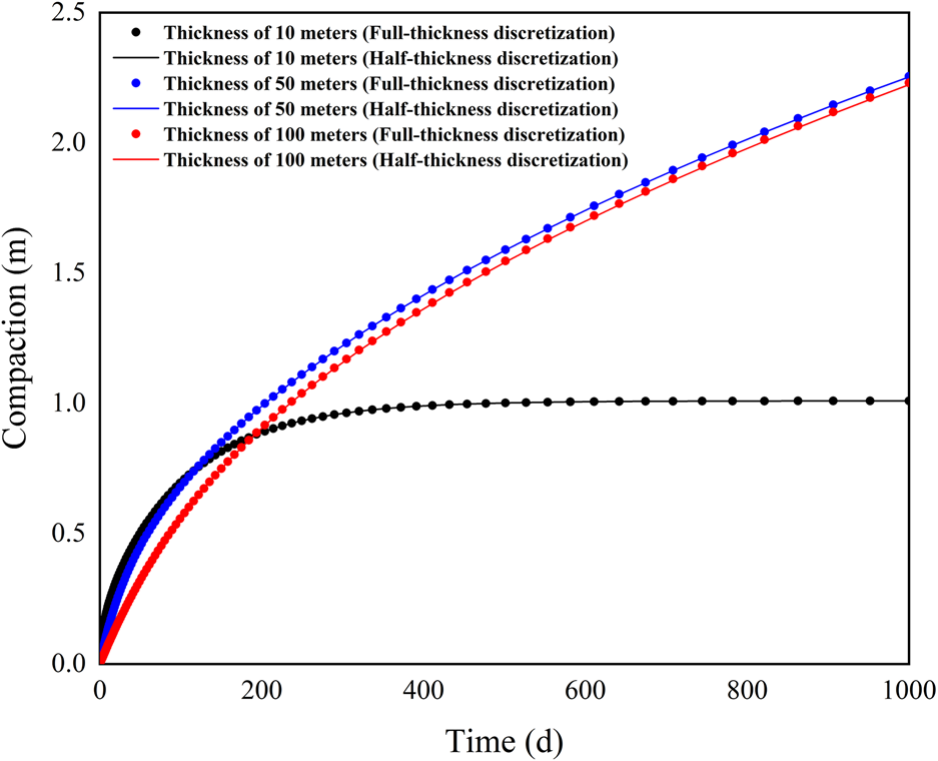
Comparison of simulated compaction for case 1.

Figure 3 shows the comparison of compaction under different interbed thicknesses. It can be observed that the smaller the thickness of the compressible delay interbed, the greater the initial rate of compaction during the stress period. The compaction is greatest for the 10 m thick interbed before 120 days, followed by the 50 m thick interbed. As compaction continues, the compaction of the thinner interbed tends to stabilize. In the scenario with a thickness of 10 m, the maximum compaction is approximately 1.0098 m. By 1000 days, the compaction process for the 50 m thick interbed slows down, with a compaction of about 2.2549 m using the full-thickness discretization method and approximately 2.2532 m using the half-thickness discretization method, resulting in a difference of 0.0017 m. At this time, the compaction process for the 100 m thick interbed is still ongoing, with subsidence amounts of 2.2301 m and 2.2231 m for the full-thickness and half-thickness discretization methods, respectively, showing a difference of 0.007 m. Table 3 presents the equilibrium table of the groundwater system under three scenarios. The increase in interbed thickness only enhances the non-elastic compaction discharge, which then flows out through the constant head cells. The compaction release amount of the interbed and the ground subsidence amount remain numerically consistent. A relative deviation metric is introduced to quantify the computational differences between the two methods. This metric is calculated as the difference between the consolidation water release from the full-thickness discretization method and the corresponding release from the half-thickness method, divided by the water release from the full-thickness method, expressed as a percentage. A positive value indicates that the full-thickness method yields higher values. The relative deviations of the compaction release amounts under different thicknesses are 0.0033%, 0.0754%, and 0.314%, respectively. This indicates that the difference between the two methods is positively correlated with the thickness of the interbed, though the impact is minimal. Even in the extreme scenario with a thickness of 100 m, the deviation between the two methods is only 0.314%. These results demonstrate that the half-thickness discretization method not only has high accuracy but also exhibits good applicability.

**Table 3:** Groundwater system balance table.

Source and sink terms (Unit: m^3^)	Thickness of 10 m		Thickness of 50 m		Thickness of 100 m	
	Full-thickness discretization method	Half-thickness discretization method	Full-thickness discretization method	Half-thickness discretization method	Full-thickness discretization method	Half-thickness discretization method
Elastic drainage of interbed	0.0000	0.0000	0.0000	0.0000	0.0000	0.0000
Inelastic drainage of interbed	1.0098	1.0098	2.2549	2.2532	2.2301	2.2231
Constant Head Inflow	0.0000	0.0000	0.0000	0.0000	0.0000	0.0000
Elastic storage of interbed	0.0000	0.0000	0.0000	0.0000	0.0000	0.0000
Inelastic storage of interbed	0.0000	0.0000	0.0000	0.0000	0.0000	0.0000
Constant head outflow	1.0098	1.0098	2.2549	2.2532	2.2301	2.2231
Storage variable	0.0000	0.0000	0.0000	0.0000	0.0000	0.0000
Absolute error	0.0000	0.0000	0.0000	0.0000	0.0000	0.0000
Relative error	0.0001%	0.0000%	0.0000%	0.0000%	0.0000%	0.0000%
Relative deviation of different methods	0.0033%		0.0754%		0.3140%	

**Fig. 4:**
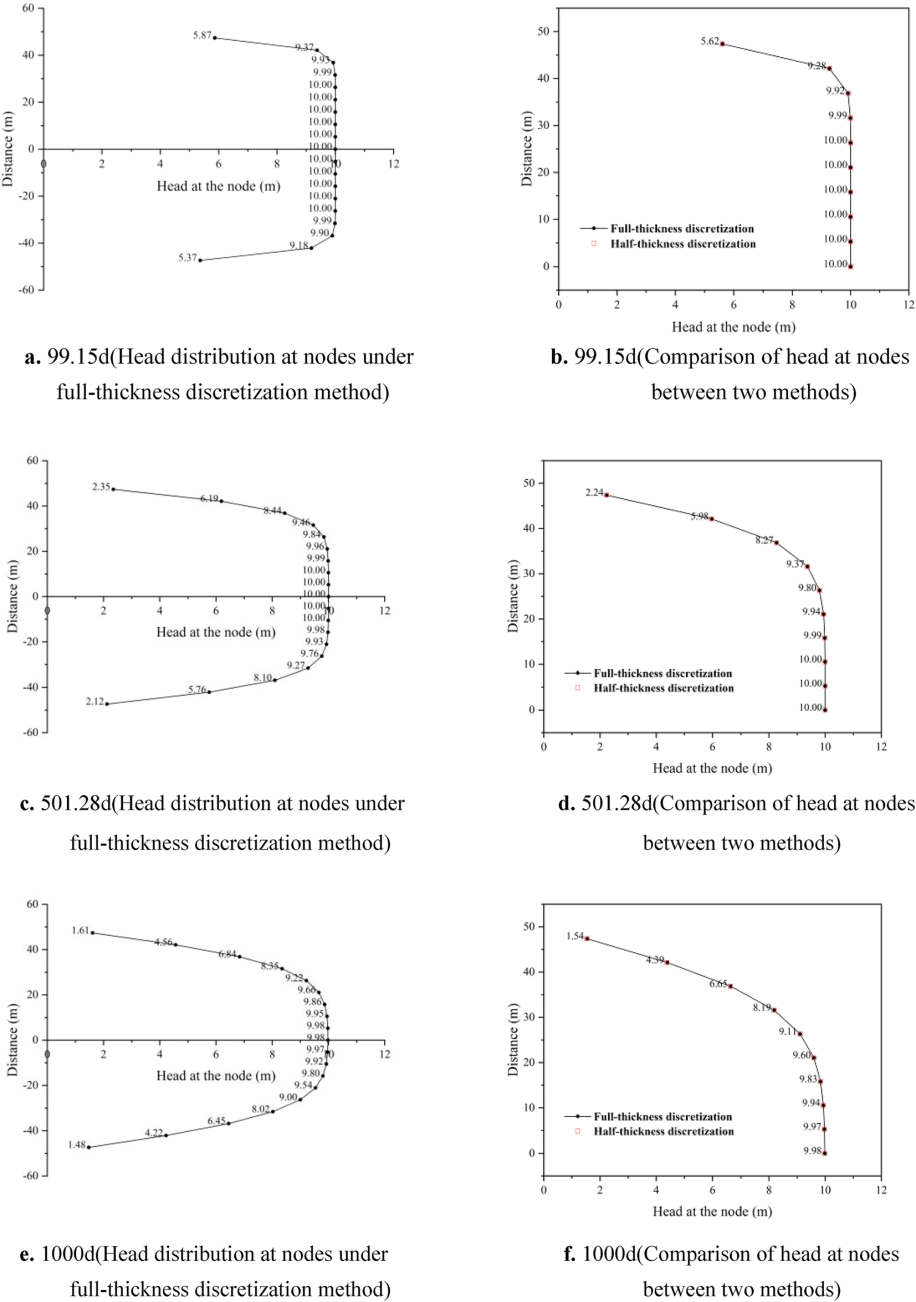
Comparison of head distribution at nodes between full-thickness discretization and half-thickness discretization methods (a, c, e represents the head distribution at 99.15 d, 501.28 d, and 1000 d under the full-thickness discretization method, respectively. b, d, f represents the comparison of head at nodes between the two methods at 99.15 d, 501.28 d, and 1000 d, respectively).

Figure 4 shows the comparison of hydraulic heads between full-thickness discretization nodes and half-thickness discretization nodes under the scenario of a 100 m thick compressible delay interbed. In panels a, c, and e, the hydraulic head distribution of all discretization units in the delay interbed is represented at 99.15 days, 501.28 days, and 1000 days, respectively. As the interbed undergoes continuous compaction, the hydraulic head values of the discretization units gradually decrease from the outside towards the center, with greater changes in hydraulic head occurring further away from the center point of the interbed. At 99.15 days, the hydraulic head value at the center node rapidly decreases to 10 m. The effective stress gradually increases from top to bottom in the interbed, resulting in a more pronounced decrease in the hydraulic head values of the units below the central point. By 1000 days, the hydraulic head values of all discretization units further decrease to varying degrees. The average hydraulic head values of all symmetrical nodes are summed and compared with the half-thickness discretization method, as shown in panels b, d, and f. It can be observed that the water level values of nodes at the three time points are basically consistent. Although the full-thickness discretization method shows noticeable differences in water levels at the outer discretization nodes during the initial stress period, the averaged results are generally consistent with those of the half-thickness discretization method. This indicates that the average hydraulic head value calculated by the half-thickness discretization method is reasonable.

### Case 2

**Fig. 5:**
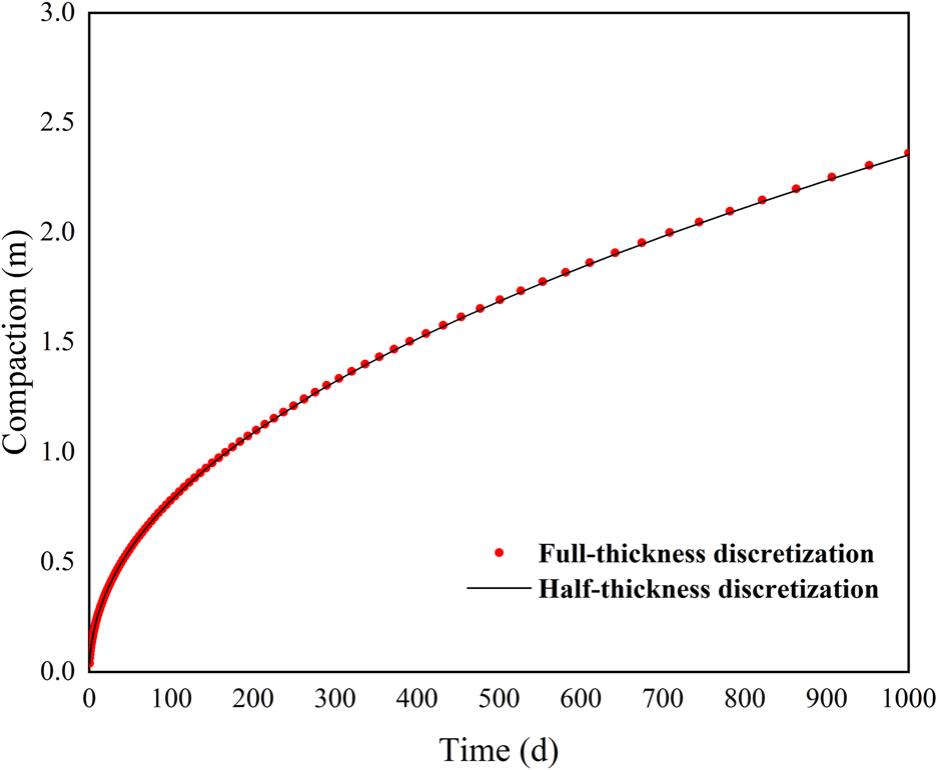
Comparison of simulated compaction for case 2.

Figure 5 shows the compaction calculation results for example 2. By 1000 days, the compaction calculated using the full-thickness discretization method is 2.3612 m, while the compaction calculated using the half-thickness discretization method is 2.3531 m, resulting in a difference of only 0.0081 m. Compared to example 1, where the compressible delay interbed thickness is 100 m, the number of discretization units has increased more than 50 times and the initial hydraulic head has increased by 10 times, yet the difference in subsidence amounts is only 1.1 mm. This indicates that the number of discretization units has a minimal impact on the calculation results of the half-thickness discretization method. Table 4 presents the equilibrium table of the groundwater system. The interbed exhibits both elastic and non-elastic compaction. Influenced by the rate of water storage, the non-elastic compaction is significantly greater than the elastic compaction. The final groundwater equilibrium results are also quite similar, with the relative deviation of the interbed discharge between the two methods being only 0.3430%.

**Table 4:** Groundwater system balance table.

Source and sink terms (unit: m^3^)	Full-thickness discretization method	Half-thickness discretization method
Elastic drainage of interbed	0.1008	0.1004
Inelastic drainage of interbed	2.2604	2.2527
Constant head Inflow	0.0000	0.0000
Elastic storage of interbed	0.0000	0.0000
Inelastic storage of interbed	0.0000	0.0000
Constant head outflow	2.3612	2.3531
Storage variable	0.0000	0.0000
Absolute error	0.0000	0.0000
Relative error	0.0000%	0.0000%
Relative deviation of different methods	0.3430%	

**Fig. 6:**
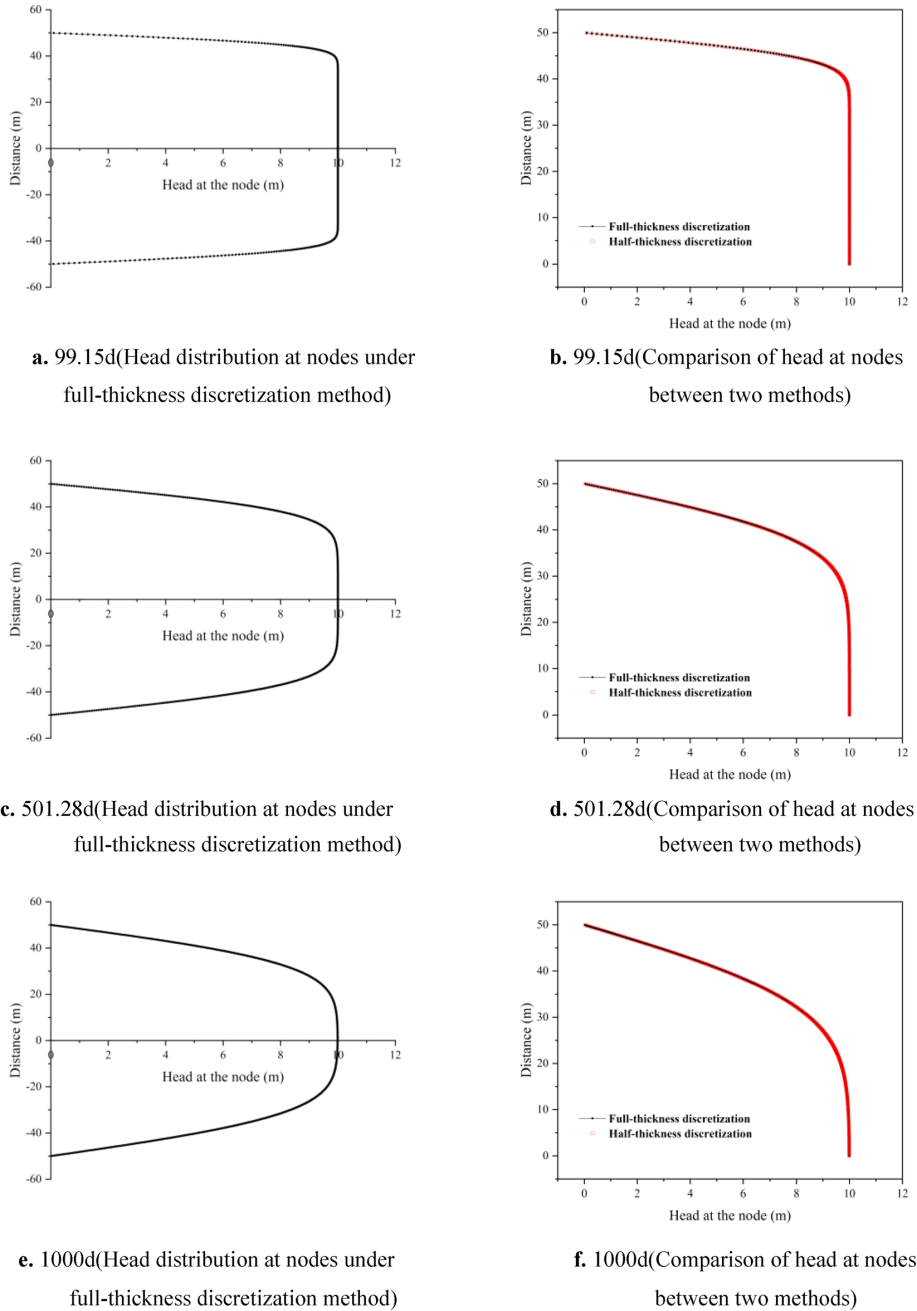
Comparison of head distribution at nodes between full-thickness discretization and half-thickness discretization methods (a, c, e represents the head distribution at 99.15 d, 501.28 d, and 1000 d under the full-thickness discretization method, respectively. b, d, f represents the comparison of head at nodes between the two methods at 99.15 d, 501.28 d, and 1000 d, respectively).

In Fig. 6, panels a, c, and e illustrate the hydraulic head distribution at all nodes under the full-thickness discretization method, corresponding to the time points of 99.15 days, 501.28 days, and 1000 days, respectively. By 99.15 days, the hydraulic head at the edge nodes of the interbed has decreased to the order of 10^–1^. Compared to the discretization method used in example 1 with 19 nodes, the hydraulic head distribution across the entire interbed is more continuous. The initial rate of hydraulic head dissipation is faster; however, as time progresses, it exhibits a similar variation process, with hydraulic head values gradually decreasing from the edge of the interbed towards the center, and the rate of decline becoming more gradual. Panels b, d, and f show the comparison of half-thickness discretization units at the three time points. The hydraulic head values under the full-thickness discretization method are also averaged results. It can be observed that the hydraulic head distribution is generally consistent. Theoretically, a greater number of nodes leads to more accurate results for the diffusion equation, but it also increases memory consumption for model calculations, affecting computational efficiency. The results indicate that the half-thickness discretization method achieves a calculation accuracy comparable to the full-thickness discretization method in scenarios with extreme thickness and a large number of discretization units. It not only improves computational efficiency but is also theoretically justified.

### Case 3

**Fig. 7:**
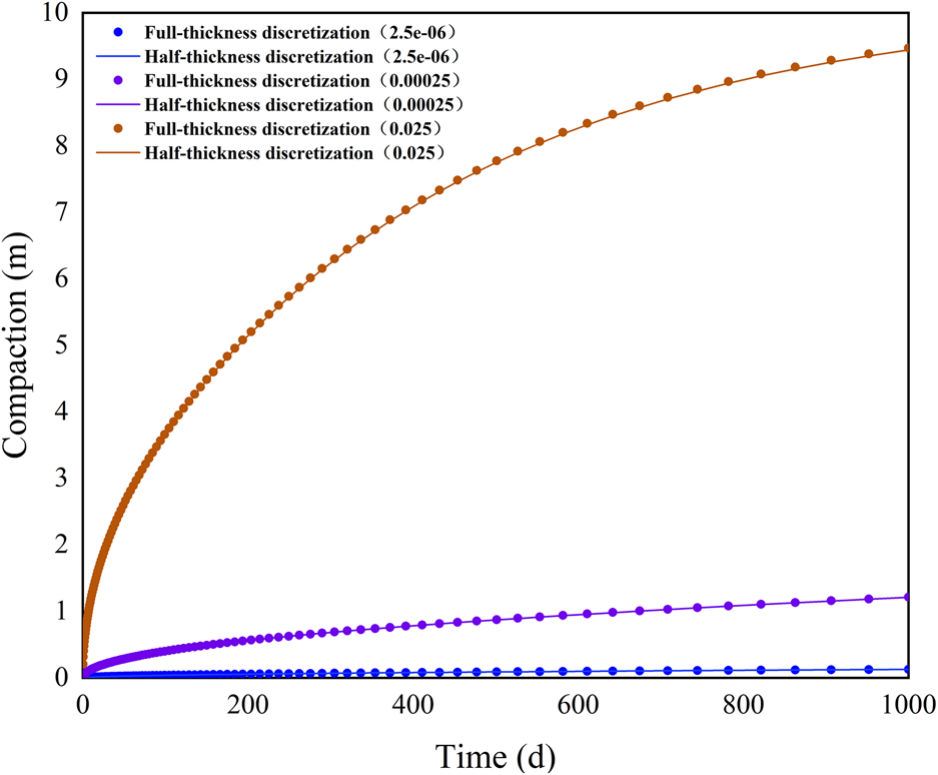
Comparison of simulated comparison for case 3.

Figure 7 presents a comparison of simulated compaction under different vertical hydraulic conductivities scenarios for the compressible delay interbed. It can be observed that as the vertical hydraulic conductivity increases, the subsidence significantly increases; however, the difference between the two methods remains minimal. By 1000 days, under the scenario with a vertical hydraulic conductivity of 2.5e−06, the compaction calculated using the full-thickness and half-thickness discretization methods are 0.1274 m and 0.1269 m, respectively, with a difference of only 0.5 mm. When the vertical hydraulic conductivity is 0.00025, the comparison under the full-thickness discretization method is 1.2154 m, while under the half-thickness discretization method it is 1.2110 m. At this point, the compaction has increased by approximately 9.5 times, and the difference between the two methods has only increased by 3.9 mm. When the vertical hydraulic conductivity is 0.025, the compaction calculated using the full-thickness and half-thickness discretization methods are 9.4716 m and 9.4419 m, respectively, resulting in an increased deviation of 0.0297 m, but the relative deviation is only 0.3136%. Table 5 presents the equilibrium table of the groundwater system, where it can be observed that as the vertical hydraulic conductivity increases, the elastic discharge of the interbed changes only slightly. The decrease in hydraulic head primarily leads to a significant increase in non-elastic discharge. Additionally, the maximum relative deviation across the three scenarios is also less than 0.4%. These results indicate that even under substantial subsidence conditions, the deviation between the half-thickness and full-thickness discretization methods remains minimal, and the rate of hydraulic head dissipation within the interbed does not affect the accuracy of the half-thickness discretization method.

**Table 5:** Groundwater system balance table.

Source and sink terms (unit: m^3^)	2.5e−06		0.00025		0.025	
	Full-thickness discretization method	Half-thickness discretization method	Full-thickness discretization method	Half-thickness discretization method	Full-thickness discretization method	Half-thickness discretization method
Elastic drainage of interbed	0.0360	0.0359	0.1013	0.1009	0.0972	0.0968
Inelastic drainage of interbed	0.0914	0.0910	1.1141	1.1101	9.3744	9.3451
Constant head inflow	0.0000	0.0000	0.0000	0.0000	0.0000	0.0000
Elastic storage of interbed	0.0000	0.0000	0.0000	0.0000	0.0000	0.0000
Inelastic storage of interbed	0.0000	0.0000	0.0000	0.0000	0.0000	0.0000
Constant head outflow	0.1274	0.1269	1.2154	1.2110	9.4716	9.4419
Storage variable	0.0000	0.0000	0.0000	0.0000	0.0000	0.0000
Absolute error	0.0000	0.0000	0.0000	0.0000	0.0000	0.0000
Relative error	0.0014%	0.0000%	0.0001%	0.0000%	0.0000%	0.0000%
Relative deviation of different methods	0.3925%		0.3620%		0.3136%	

### Comparison of computation time and memory usage

To quantitatively evaluate the improvement in computational efficiency of the half-thickness discretization method compared to the full-thickness discretization method, a comparative analysis was conducted based on Case 2. The number of nodes was set to 100,001 for the full-thickness method and 50,001 for the corresponding half-thickness method. Both approaches were implemented using COMUS, and the total runtime and memory usage of the models were recorded. The results are presented in Table 6. Two metrics—absolute difference and relative difference—were introduced to assess the efficiency gap between the two methods. The absolute difference reflects the numerical gap between results, while the relative difference, defined as the percentage of the absolute difference relative to the full-thickness method result, indicates the extent of performance improvement. The comparison results show that the half-thickness discretization method reduces computation time by 46.2303%, demonstrating a significant advantage in efficiency. Additionally, it reduces memory usage by 6.9 MB, which is 13.6364% less than that of the full-thickness method. It is worth noting that the total memory consumption of the model includes not only the interbed calculations but also other computational modules. Therefore, the actual memory savings from the half-thickness method may be even more substantial in practice. In routine simulations, the model may contain tens of thousands of cells. Taking this case as an example, under the full-thickness method, each additional node requires approximately 0.000138 MB of memory. If the model contains 50,000 cells, each with one interbed discretized into 41 nodes, the half-thickness method could save at least 138 MB of memory. Thus, the proposed method offers significant practical benefits in reducing computational resource consumption.

**Table 6:** Comparison of computation time and memory usage.

Number of nodes, Computation time (s), and Memory usage (MB)	Full-thickness discretization method	Half-thickness discretization method
Number of nodes	100,001	50,001
Computation time	81.453	43.797
Memory usage	50.6	43.7
Absolute difference in computation time	37.656	
Relative difference in computation time	46.2303%	
Absolute difference in memory usage	6.9	
Relative difference in memory usage	13.6364%	

## Discussion and conclusions

This study focuses on the grid discretization problem of compressible delay interbeds in simulating subsidence while considering changes in aquifer storage characteristics. Given that the MODFLOW-CSUB package sacrifices computational efficiency to account for variations in vertical stress in compressible delay interbed, this research proposes a half-thickness discretization method that differs from the discretization approach used in the CSUB package. This method is based on the characteristic of linear distribution of geostress along the vertical direction of the delay interbed. When calculating compressible delay interbeds, this approach can consider changes in stress and storage characteristics like the CSUB package, while also reducing the computational process by half during one-dimensional diffusion equation calculations, theoretically offering higher efficiency while maintaining good accuracy.

To validate the newly proposed half-thickness discretization calculation method for interbeds with delay that considers changes in storage characteristics, three representative test cases were set up based on MODFLOW-CSUB example 2. These cases account for variations in the number of discretization units, different interbed thicknesses, different hydraulic heads, and vertical hydraulic conductivities. The results indicate that compared to the full-thickness discretization method for interbeds with delay, the half-thickness discretization method still provides accurate compaction calculation results without the need to discretize the entire interbed for solving. The averaged hydraulic head values for each discretization unit align with reality, demonstrating that this method is effective. Next, the calculation accuracy of the half-thickness discretization method is minimally affected by interbed thickness, vertical hydraulic conductivity, and the number of discretization units, demonstrating strong applicability. This method is suitable even for scenarios involving thick interbeds with delay and significant subsidence. In cases with a larger number of discretization units, the results are generally consistent with those of the full-thickness discretization method. The comparison results of computation time and memory usage show that the half-thickness discretization method reduces computation time by 46.2303% and memory usage by 13.6364%, demonstrating a clear advantage in computational efficiency. These findings indicate that the proposed method significantly improves the efficiency of land subsidence modeling while maintaining a high level of accuracy.

Moreover, the proposed half-thickness discretization method of delay interbed is easy to implement and has a rational basis validated by simple scenarios. This method is particularly useful in regions with broad coverage, complex geological conditions, and significant subsidence risks, such as the Jing-Jin-Ji Plain^22^, California’s Central Valley^23^, and Tehran Plain in Iran^24^. In large-scale subsidence prediction and risk assessments, it is often necessary to construct large, high-resolution, long-time-series groundwater-subsidence coupling models. This method can effectively reduce model runtime and hardware resource consumption, improving the feasibility of multi-scenario simulations and decision support, thus enabling more efficient and intelligent dynamic subsidence simulations and control. It should be noted that while the proposed method performs well in several typical case studies, it does have certain limitations. Specifically, the half-thickness discretization method serves as an approximate alternative to the full-thickness discretization, with the full-discretization method still having advantages in simpler scenarios. In the future, this method will be further developed and applied to more complex site conditions.

## Data Availability

Software code and data used for this work, including model files and ancillary data, are available through the GitHub repository: [https://github.com/hsq995/comus_sub.git].
